# Evaluation of low nitrogen resistance of *Avena sativa* germplasm during the seed germination period

**DOI:** 10.1016/j.heliyon.2024.e40765

**Published:** 2024-11-28

**Authors:** Jing Pan, Zeliang Ju, Xiang Ma, Lianxue Duan, Zhifeng Jia

**Affiliations:** Key Laboratory of Superior Forage Germplasm in the Qinghai-Tibetan Plateau, Qinghai Academy of Animal Husbandry and Veterinary Sciences, Qinghai University, Xining, China

**Keywords:** Low-nitrogen stress, Oat, Physiological response, Comprehensive assessment

## Abstract

Nitrogen (N) plays a crucial role in forage yield. However, excessive application of N fertilizers in agricultural production not only increases the production cost but also leads to serious environmental problems. Therefore, mining low-N tolerant oat germplasm is important for the sustainable development of grass and pasture. Relevant physiological indices of five oat germplasm were measured and analyzed in the seedling leaves under low-nitrogen culture conditions, aiming to identify important physiological indices suitable for screening low-nitrogen-tolerant oat and to rank the low-nitrogen-tolerance ability of each oat variety. The results showed that the physiological indexes of oats under low-nitrogen conditions were significantly different from those under normal culture conditions, and the total root length, total nitrogen, photosynthesis index, and NR enzyme activity of leaves in the five oat germplasm showed a decreasing trend, while the proline, MDA, SOD, POD, and GS enzyme activities showed an increasing trend, but the magnitude of changes in the physiological indexes varied among different varieties. By analyzing the physiological indexes, the photosynthetic index and antioxidant enzyme activities were found to be important in assessing the ability of oats to tolerate low nitrogen and therefore can be used as important indexes for screening oats for low nitrogen tolerance. The results of the comprehensive analysis showed that the order of low-N tolerance of each variety was as follows: Qingyongjiu 035> Jiayan No.2> Qingyin No.2> ZNY255> ZNY256, and the results of the present study provide a reference for the further large-scale screening of low-N-tolerant oat resources and the selection of varieties.

## 1 Introduction

Nitrogen (N), one of the essential elements in plants, plays a vital role in plant growth and development and is the basis of agricultural production practices [1]. The current practical application of N fertilizer is the most direct way to promote plant growth and crop yield [2]. As the global population rapidly increases, so does the demand for N fertilizer in agriculture, and the mass production and application of N fertilizer have become common agricultural techniques used to increase crop yields [3]. According to statistics, the global annual output of N fertilizer used to improve crop yields is more than a million. In contrast, the production of N fertilizer consumes 1%–2% of global energy, but the effective use of N fertilizer is less than 50 % [4,5]. In agricultural production, remaining N fertilizer is converted into nitrous oxide (the main component of greenhouse gases) and emitted into the atmosphere, leading to global warming. China is a country with high global demand for N fertilizer, but its utilization rate of N fertilizer is far below the worldwide average [6]. As the global population grows and the demand for meat, eggs, milk and other meaty foods increases, so does the demand for the pasture grasses that livestock eat. Improving the nitrogen use efficiency of pasture grasses and effectively reducing the amount of nitrogen fertilizer applied, thereby reducing pollution and environmental impacts, has become an urgent issue.Therefore, improving the N utilization efficiency of pasture grasses without consuming more N fertilizer, and cultivating pasture grasses with high N utilization efficiency or high tolerance to low-N levels are of great significance for the sustainable development of the pasture industry.

As an annual grain-feeding crop, oat (*Avena sativa* L.) belongs to the Gramineae family and is cultivated in 42 countries worldwide [7]. Oat is also widely grown in the North China Plain, Northwest Plateau, and Northeast China due to its preference for cool climates, high-stress tolerance, high forage value, and livestock preference [8,9]. In addition, oat possess the characteristics of cold and drought tolerance, barrenness tolerance, and moderate salinity tolerance. Therefore, in practical production applications, leather oats are widely selected as a high-quality germplasm resource for agricultural and pastoral areas, whether as forage or as a planting target. Therefore, the study of differences in tolerance to low-N stress and efficient-N utilization among different oat varieties provides a specific research basis for the excavation of excellent genes for low-N tolerance in oat and the genetic improvement of breeding. Recent studies on the tolerance of plants under low-N environments and the excavation of genes for efficient N utilization has been carried out successively. Molecular tools such as molecular marker technology and gene chip localization have been used to identify some genes related to low-N tolerance and efficient N utilization efficiency in significant crops such as *Oryza sativa* [10], *Triticum aestivum* [11], *Zea mays* [12], and *Solanum tuberosum* [13], and screen out some low-N-tolerant germplasm. Most of the studies on low-N tolerance in oat focused on N photosynthetic efficiency [14], low-N tolerance phenotypic and physiological indexes [15], and yield and quality [16], and there have been few studies on low-N tolerance and the differences between varieties.

In this study, five different low-N-tolerant oat were selected as materials and physiological indexes, and changes in low-N tolerance and differences among varieties under low-N stress were comprehensively investigated. This study aims to explore the mechanism of low-N tolerance in oat, enrich the low N tolerant oat germplasm resources in China, provide materials for the excavation of low-N- tolerant genes in forage oat, and provide a specific theoretical basis for the innovation of germplasm resources and breeding practices in the Tibetan Plateau region.

## 2 Materials & methods

### 2.1 Plant materials and experimental design

The College of Animal Husbandry and Veterinary Science provided the five oat materials screened in the previous experiment: Jiayan No. 2, Qingyin No. 2, Qingyongjiu 035, ZNY255, and ZNY256. The relevant information of 5 oat materials is detailed in Table 1. The experiment was conducted from November to December 2023 in the artificial climate chamber of the College of Animal Husbandry and Veterinary Science, Qinghai University. Filled grain oat seeds were selected and sterilized by soaking in 75 % ethanol for 1 min, then rinsed in distilled water for germination. The seeds were evenly spread in seedling trays lined with two layers of filter paper and placed in an intelligent artificial climate chamber with a relative humidity of 55 % and a light period of 16 h to wait for germination. Before germination, appropriate amounts of distilled water were poured at appropriate times, and the well-grown seedlings were moved into a hydroponic tank filled with Hoagland's nutrient solution to ensure average growth, and the nutrient solution was replaced once every 5 days to ensure the average growth of the seedlings. Seedlings were divided into two groups: one group was treated with Hoagland nutrient solution containing 1.25 mM NH_4_NO_3_ [17] for low-N stress (LN), and the other group was cultured with Hoagland nutrient solution with 10 mM NH_4_NO_3_ normal N levels (CK) and was continuously stressed for 14 d. After 14 days of continuous cultivation, five healthy plants with uniform growth were selected from each treatment for the determination of agronomic traits. Fresh leaves of oat seedlings were cut and stored in an ultra-low temperature refrigeratorfor the determination of physiological indexes.e.

**Table 1:** Test oat material information.

Serial No.	Materials	Source
1	Jiayan No.2	China
2	Qingyin No.2	China
3	Qingyongjiu 035	China
4	ZNY 255	China
5	ZNY 256	China

**Table 2:** Comprehensive indicators and contribution rates.

Variable	Principal component 1	Principal component 2	Principal component 3	Principal component 4
Ci	0.968	−0.163	0.041	0.186
SS	0.917	0.006	−0.397	−0.023
Pn	−0.856	0.261	−0.394	0.21
Tr	0.841	0.131	0.523	−0.045
GS	−0.834	0.422	0.28	−0.218
Sp	0.823	−0.534	−0.013	0.192
RDW	0.747	0.565	0.019	−0.351
MDA	0.64	−0.503	0.551	−0.187
NR	0.616	0.396	−0.479	0.484
Gs	0.33	0.872	0.206	−0.298
SOD	−0.409	0.825	0.295	0.254
POD	0.626	0.664	−0.168	−0.372
SDW	−0.185	0.29	0.911	0.229
PH	0.293	0.65	−0.691	0.115
TN	0.46	0.458	0.541	0.534
Eigenvalues	6.938	3.904	2.958	1.2
Contributiion/%	46.252	26.03	19.717	8.001
Cumulative contribution/%	46.252	72.282	91.999	100

Table 1 Source of test material for this experiment.

Note: Sp: starch phosphorylase; NR: nitrate reductase; MDA: malondialdehyde; GS: glutamine synthetase; Tr: transpiration rate; Pn: net photosynthesis rate; Gs: stomatal conductance; Ci: inter-cellular CO_2_ concentration; SOD: superoxide dismutase; POD: peroxidase; SS: soluble sugars; PH: plant height; SDW: shoot dry weight; RDW: root dry weight; TN: total nitrogen.

Table 2 Coefficients and contribution of each composite indicator.

### 2.2 Determination of plant height、biomass and total nitrogen content in oat

Five oat seedlings were selected from each replication and plant height (plant height, PH) was measured using a straightedge. At the same time, the stems and leaves and roots were packed separately in envelope bags, killed in an oven at 105 °C for 30 min, dried at 60 °C until constant weight, and weighed to shoot dry weight (SDW) and root dry weight (RDW). Kjeldahl method [18] was used to determine the total nitrogen in plant (TN), each sample was ground and passed through a 0.25 mm sieve, about 0.1 g was taken for decoction, and then the volume was fixed to 100 mL, and about 10 mL of the sample was taken for the determination of nitrogen through the flow analyzer, and three replications were set up for each treatment and each variety.

### 2.3 Measurement of photosynthetic indicators in oat

To investigate the effect of low-N stress on photosynthesis in oat seedlings,the stomatal conductance (Gs), net photosynthetic rate (Pn), intercellular CO_2_ concentration (Ci), and transpiration rate (Tr) of leaves (second leaf from the top downwards) of oat material were determined under different treatments using the LI-6800 portable photosynthesizer (LI-COR, Lincoln, NE, USA), and each treatment was replicated three times.

### 2.4 Determination of related enzyme activities and malondialdehyde (MDA) content in oat

To investigate the effects of low-N stress on the changes of N metabolism-related enzyme activities in oat seedlings, the activities of starch phosphorylase (Sp), glutamine synthetase (GS), and nitrate reductase (NR) were determined according to the method of Nakamura et al. [17]. Once the different samples were treated, we measured the absorbance values of Sp and NR using a fully automated enzyme labeling instrument at a wavelength of 340 nm, and the absorbance values of GS were determined under a fully automated enzyme labeling instrument (Thermo Fisher, Waltham, MA, USA) at a wavelength of 540 nm.

To investigate the effects of low-N stress on antioxidant enzyme activities and MDA content in oat seedlings, superoxide dismutase (SOD) activity was determined using an N blue tetrazolium colorimetric assay [19], and peroxidase (POD) activity was determined using a guaiacol colorimetric assay [20]. MDA content was determined using the method of Savicka et al. [21]. After the samples for each index were processed, the absorbance values were read at 450 nm and 470 nm for SOD and POD, respectively, and at 532 nm and 600 nm for MDA.

### 2.5 Determination of the content of osmoregulatory substances in oat

To investigate the effects of low-N stress on the changes of osmoregulatory substances in oat seedlings, the soluble sugar content (SS) was determined by anthrone colorimetry [22], and the absorbance value was read at 620 nm after sample treatment.

### 2.6 A comprehensive evaluation of low N tolerance in oat

Five oat materials were correlated at the seedling stage, and the 15 physiological indexes measured were downscaled using principal component analysis (PCA) to screen the comprehensive indexes of low-N tolerance. Finally, the affiliation function method was used to evaluate low-N tolerance [23].

The coefficient of low-N tolerance = the average value of index/control index under low-N stress

*U(X*_*j*_*)=(X*_*j*_*-X*_*min*_*)/(X*_*max*_*-X*_*min*_*)*,*j* = 1, 2, 3, …, n

In the formula, *X*_*j*_ represents the *j* comprehensive index; *X*_min_ represents the minimum value of the *j* comprehensive index; *X*_max_ represents the maximum value of the *j* comprehensive index.

$$W_{j}=P_{j}/\sum_{j=1}^{n}P_{j},j=1,2,3,\ldots ,n$$

In the formula, *W*_*j*_ indicates the importance of the *j* comprehensive index in all total indicators, that is, weight, and *P*_*j*_ is the contribution rate of the *j* comprehensive index of each genotype.

$$D=\sum_{j=1}^{n}\left[U\left(X_{j}\right)*\text{Wj}\right],j=1,2,3,\ldots ,n$$

In the formula, the *D* value is the comprehensive evaluation value of the low-N tolerance of each genotype under low-N stress.

### 2.7 Data processing and statistical analysis

All data in this experiment were collated using Excel 2020, and were analyzed by ANOVA, PCA, and correlation analysis using SPSS 22.0 software.

## 3 Results

### 3.1 Effect of low-N stress on morphological indicatorsand total nitrogen content in oat

**Fig. 1:**
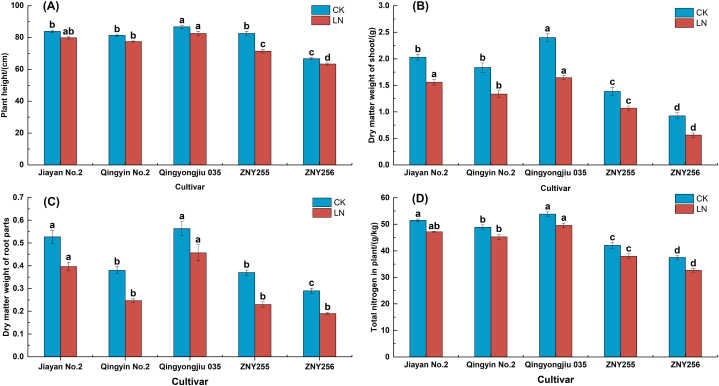
Morphological indicators and total nitrogen content of oats under low nitrogen stress. (A) Plant height. (B) Aboveground dry weight. (C) Below-ground dry weight. (D) Total plant nitrogen content.

There were highly significant differences (*P < 0.05*) in agronomic traits and plant nitrogen content of oat varieties under low nitrogen stress treatment conditions. The ranges of the four traits related to nitrogen efficiency differed significantly at both nitrogen levels (Fig. 1). The indicators of plant length (Fig. 1-A), dry weight of above-ground part (Fig. 1-B), dry weight of below-ground part (Fig. 1-C) and total plant nitrogen content (Fig. 1-D) increased with increasing nitrogen levels. In particular, the extent of variation in aboveground part dry weight was the greatest among oat varieties for each morphological trait index at both nitrogen treatment levels, which is favorable to show differences among germplasm.

### 3.2 Effect of low-N stress on photosynthetic parameters in oat

**Fig. 2:**
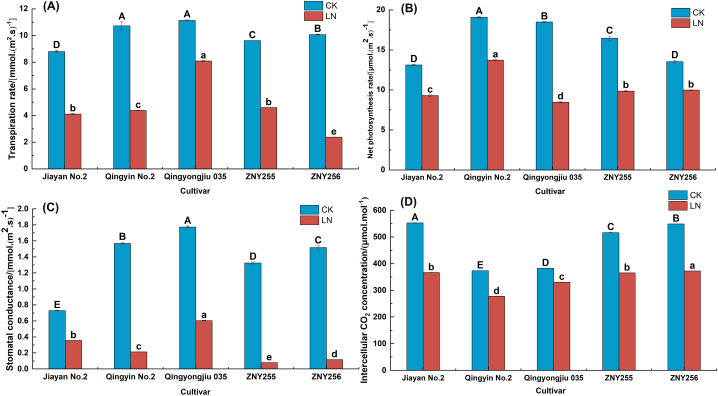
Photosynthetic characteristics of oat leaves under different low-nitrogen stress times.(A) Transpiration rate. (B) Net photosynthetic rate. (C) Stomatal conductance. (D) Inter-cellular CO_2_ concentration.

Under low-N stress, the Pn of all five materials decreased, while the difference in Pn of each material at different time points was significant (*P < 0.05*). At 14 days of low N stress, the Pn of the five materials (Jiayan No. 2, Qingyin No. 2, Qingyongjiu 035, ZNY255, and ZNY256) decreased to different degrees (Fig. 2-B), with Qingyongjiu 035 decreasing the most (54.21 %). Among the photosynthetic coefficients, the stomatal conductance of Qingyongjiu 035 decreased the most (Fig. 2-C), indicating that the photosynthetic index affecting the normal growth of green permanent under low nitrogen stress was stomatal conductance. Under low-N stress, the Tr and Gs of the five materials decreased with the extension of stress time (Fig. 2-A, D), and the trends were the same as that of the Pn, with significant differences among the five materials (*P < 0.05*). The decrease in Tr and Gs was greater in ZNY256 compared to the control than in the other materials. (*P < 0.05*). The Ci of the five materials decreased significantly with the treatment time, with that of Jiayan No. 2 decreasing by 33.71 % compared with the CK group, which was significantly (*P < 0.05*) different from that of the other materials.

### 3.3 Effects of low-N stress on the activities of enzymes related to N metabolism and MDA content in oat

**Fig. 3:**
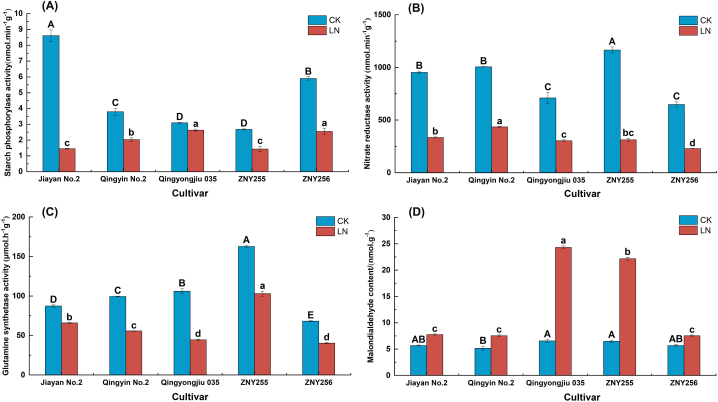
Changes in enzyme activities related to oats' nitrogen metabolism under low-nitrogen stress. (A) Starch phosphorylase. (B) Nitrate reductase. (C) Glutamine synthetase. (D) Malondialdehyde content.

Under low-N stress, the Sp content of the test swiftlet germplasm showed overall different degrees of decrease. As can be seen in Fig. 3-A, the Sp activity of Jiayan No. 2 decreased the most (83.12 %), and Qingyongjiu 035 decreased the least (15.09 %). The NR activities of the test oat materials under low-N stress treatments all decreased to different degrees (Fig. 3-B), with smaller decreases in Qingyin No. 2 and Qingyongjiu 035 (56.76 % and 57.21 %, respectively), and the most significant reduction in ZNY255 (73.14 %). GS showed a decreasing trend compared to the control (Fig. 3-C). The difference was significant (*P < 0.05*), with the most minor decrease (23.37 %) in Jiayan No. 2's GS activity, followed by ZNY255 and ZNY256, which showed a reduction of 36.68 and 40.87 %, respectively, and the most significant decrease (58.00 %) in Qingyongjiu 035. MDA content showed an increasing trend under low-N stress (Fig. 3-D), with the smallest increase in ZNY256 (32.87 %), and more significant increases in ZNY255 and Qingyongjiu 035 (242.35 % and 270.94 %, respectively). A comparison of the MDA results between the CK group and the low-N stress group revealed that Qingyongjiu 035 had the best content when no stress was applied, and its MDA content in the body was also at a higher level among the low-N stress groups after low N stress.

### 3.4 Effect of low-N stress on the antioxidant enzyme activities in oat

**Fig. 4:**
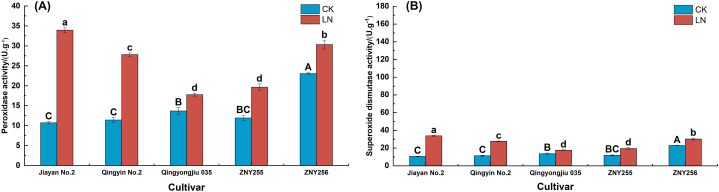
Changes in reactive oxygen species in oat under low-nitrogen stress. (A) Peroxidase activities. (B) Superoxide dismutase.

The POD activity (Fig. 4-A) and SOD activity (Fig. 4-B) of the five oat cultivars showed different degrees of increase under low nitrogen stress conditions (*P < 0.05*). From Fig. 4A, it can be seen that Jiayan No.2 had the greatest increase in POD activity, followed closely by Qingyin No.2. In contrast, Qingyongjiu 035 showed the smallest increase.Under low-N stress, the most significant increase in SOD activity was observed in Jiayan No. 2 (216.30 %), while smaller increases were observed in Qingyongjiu 035 and ZNY256 (29.95 % and 31.67 %, respectively).

### 3.5 Effects of low-N stress on the content of osmotic regulatory substances in oat

**Fig. 5:**
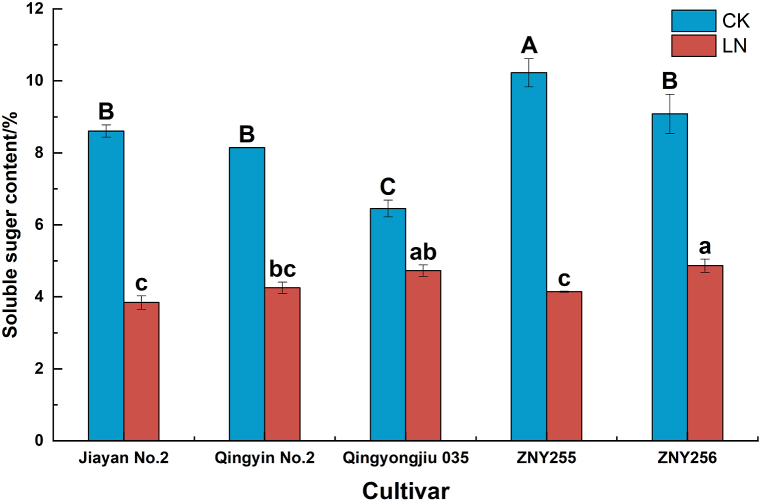
Changes in the content of osmoregulatory substances in oat under low-nitrogen stress.

Under low-N stress, the SS content of all the tested varieties decreased, but the degree of decrease in SS content differed significantly (*P < 0.05*) among the materials (Fig. 5). Among them, ZNY255 showed the most significant decrease (59.52 %), followed by Jiayan No. 2 (55.35 %), Qingyin No.2 and ZNY256 showed more consistent decreases (47.49 % and 46.46 %), and Qingyongjiu 035 showed the most minor decline (26.77 %).

### 3.6 Effects of low-N stress on the content of osmotic regulatory substances in oat

**Fig. 6:**
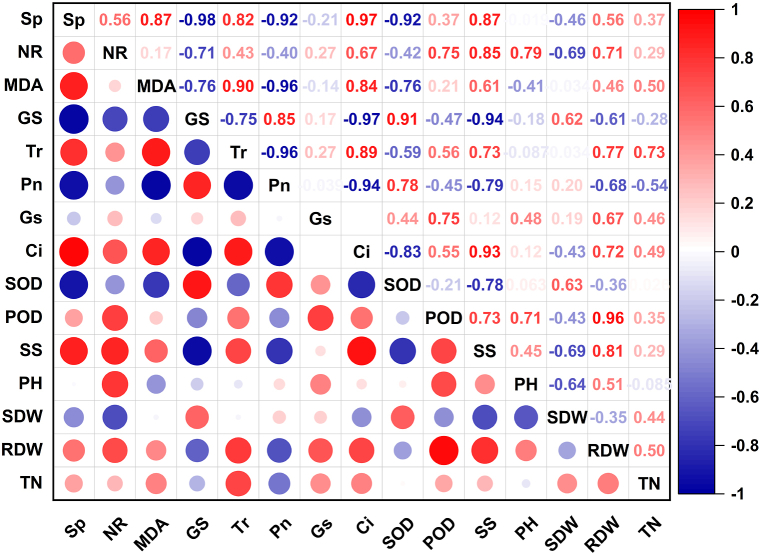
Correlation analysis of various indexes of five oat materials. Note: Different colors represent different levels of relevance. Sp: starch phosphorylase; NR: nitrate reductase; MDA: malondialdehyde; GS: glutamine synthetase; Tr: transpiration rate; Pn: net photosynthesis rate; Gs: stomatal conductance; Ci: inter-cellular CO_2_ concentration; SOD: superoxide dismutase; POD: peroxidase; SS: soluble sugars; PH: plant height; SDW: shoot dry weight; RDW: root dry weight; TN: total nitrogen.

In this study, 15 physiological indices of five oat materials were correlated (Fig. 6). The results showed that 11 pairs of traits were significantly correlated (*P < 0.05*), among which the RDW coefficient was significantly positively correlated with the POD coefficient (0.970, *P < 0.01*), and the GS coefficient was significantly negatively correlated with the Sp coefficient with a correlation coefficient of −0.958. The Pn coefficient was negatively correlated with the MDA and Tr coefficients with a correlation coefficient of −0.935 and −0.955, respectively. The Ci coefficient was also significantly negatively correlated with the GS coefficient (−0.905, *P < 0.05*). Therefore, using only one or a few physiological indexes to evaluate the tolerance of oat to low nitrogen is not very reliable, and a comprehensive analysis is necessary.

The physiological indicators of the five oat materials were subjected to PCA, and the analysis showed an overlap of information between the physiological indicators. Therefore, PCA was used to analyze the data in this experiment. As a result, four principal components were extracted with variance contributions of 46.25 %, 26.03 %, 19.72 %, and 8.00 %, respectively. After a comprehensive analysis of the physiological indexes, the results showed that the coefficients of Ci, Suger, and Tr were higher for principal component 1 (0.968, 0.917, and 0.841, respectively). The results showed that the Ci, Suger, and Tr coefficients of principal component 1 were high (0.968, 0.917 and 0.841, respectively). The coefficient of MDA content of principal component 3 was significant (0.551). The TN index of principal component 4 was high (0.534). The results showed that the trends of changes in the photosynthetic index and antioxidant enzyme activities of oats under low nitrogen stress were obvious and significant. These physiological indexes can be used as the main physiological indexes to cope with low nitrogen stress in oat seedling stage.

Using the affiliation function for a comprehensive evaluation, plant resistance can be effectively measured and accurately reflect the resistance strength of the test materials. Based on the affiliation function method, four principal components were calculated for each oat material, and the weights of the three main components were then calculated as 0.46, 0.26, 0.20, and 0.08, respectively. Finally, the comprehensive evaluation value of salt tolerance (D-value) of each oat material was calculated and ranked in order of the total evaluation value (Table 3). The order of strong to weak low-N tolerance was Qingyongjiu 035> Jiayan No. 2> Qingyin No. 2> ZNY255> ZNY256.

**Table 3:** The comprehensive indicator values, index weight, membership function value, D value, and comprehensive evaluation of 5 oat materials seeding.

Materials	Principal component 1	Principal component 2	Principal component 3	Principal component 4	U (X1)	U (X2)	U (X3)	U (X4)	D values	Rank
Jiayan No.2	−1.4	3.03	0.79	−0.91	0.02	1.00	0.70	0.03	0.41	2
Qingyin No.2	−0.34	0.38	−0.48	1.69	0.19	0.50	0.43	1.00	0.38	3
Qingyongjiu 035	4.63	−0.08	0.02	0.06	1.00	0.41	0.54	0.39	0.71	1
ZNY 255	−1.36	−2.26	2.17	0.15	0.03	0.00	1.00	0.43	0.24	4
ZNY 256	−1.53	−1.07	−2.5	−1.00	0.00	0.22	0.00	0.00	0.06	5
Weight					0.46	0.26	0.20	0.08		

Table 3. The comprehensive indicator values, index weight, membership function value, *D* value, and comprehensive evaluation of five oat seedings.

## 4 Discussion

Crops have complex physiological response mechanisms to low-N stress, and there are differences in tolerance to low-N stress among different genotypes. Currently, research on nitrogen fertilizer reduction in oats mainly focuses on agronomic trait analysis and indicators such as nitrogen accumulation in field experiments to reveal the response of different oat varieties to nitrogen [17]. However, this approach is constrained by a variety of factors such as seasonality and weather, making it difficult to achieve the goal of screening oat varieties tolerant to low N in large quantities. In contrast, the method of screening low N tolerant varieties of oats by hydroponics at the seedling stage has the advantages of being rapid, effective, and at a significantly reduced cost. In this study, we measured and analyzed the physiological indices of five oat materials under low-N stress conditions. We observed significant differences in physiological indices of different oat germplasm under low-N treatments and their tolerance to low-N stress.

Plants reduce soil NO_3_^−^ to NH_4_^+^ and assimilate it through a series of N metabolism reactions to produce N-containing organic matter that plants can absorb to maintain average growth and development. This process involves regulating several N metabolism-related enzymes [24]. The performance of N metabolism-related enzyme activities reflects the N-metabolizing capacity of plants. NR is the primary rate-limiting enzyme for N metabolism in plants, which can catalyze the reduction of NO_3_^−^ absorbed by plants to NH_4_^+^. Therefore, determining NR activity is more critical to studying the response of plants to low-N stress [25]. GS is one of the crucial enzymes for the assimilation of ammonia in plants, and it has been shown that high expression of GS improves the efficiency of N utilization in plants under environmental conditions of insufficient N supply [26]. Sp has an essential regulatory role in plants. It can regulate N metabolism in plants and store most of the organic N [27]. In this experiment, the Sp, GS, and NR activities of all test materials showed a decreasing trend, which is consistent with previous studies on the activities of enzymes related to N metabolism during the seedling stage of maize under low-N stress [28]. Moreover, we noticed that the Sp and NR activities of Jiayan No. 2 decreased significantly under low-N stress, indicating that the ammonia conversion capacity of this variety is susceptible to low-N stress.

It has been shown that plant adaptation to abiotic stresses is achieved by regulating physiological and biochemical responses. In this process, the content of osmoregulatory substances can be used as an indicator to assess plant resistance [29]. Increasing organic matter content, such as SS, helps maintain the water absorption capacity of plant leaves, thereby delaying the leaf senescence process. Under low-N stress, the lack of exogenous N supply to the plant leads to a decrease in N compounds absorbed, thus causing changes in osmoregulatory substances [30]. In plants, nitrogen metabolism and carbon metabolism interact with each other and jointly affect plant growth and development. Soluble sugars are an important source of carbon in plants, and glycolysis has an irreplaceable role in the production of carbon skeleton, which helps plants cope with adverse environments [23]. Some studies have shown that low-N stress decreased SS content in maize, indicating that SS synthesis was affected by low-N stress [31]. In this study, low-N stress conditions significantly reduced the SS content of oat (*P < 0.05*). This result indicated that the ability of oat to self-synthesize N compounds was concentrated under a low-N environment, which affected the synthesis of SS in oat and weakened its water-absorbing and osmotic-regulating abilities, which ultimately led to the senescence of oat seedling leaves.

NR acts as the first enzyme for NO_3-_ reduction to NO_2-_, and NR is also a limiting enzyme for nitrogen assimilation, and low nitrogen stress significantly inhibits NR activity in plants [10]. NR activity in plant leaves was affected by the reduction of nitrate content in the nutrient solution under low-N stress. It showed a significant decreasing trend [32]. The effects of low-N stress on SOD, POD, and GS enzyme activities in oat leaves showed an increasing trend, which may alleviate the tissue cell damage induced by low-N stress, scavenge reactive oxygen radicals, and enhance the N uptake and transport capacity of oat [17]. Oat leaves counteract the overload accumulation of reactive oxygen species caused by low-N stress by boosting SOD and POD enzyme activities in vivo. Plants can provide an available N source by converting inorganic N to organic N catalyzed by NH^4+^ and glutamate combined with GS [33]. Researchers have used PCA and road number function analyses to analyze the effects of rice varieties under culture conditions with different N levels and concluded that plant height and adequate tiller number can be used to identify low-N tolerance in *Oryza sativa* [34]. Some researchers studied the agronomic traits and physiological indexes of 20 sorghum varieties under low-N culture conditions and synthesized Pearson's correlation analysis, PCA, and the analysis of the affiliation function, and found that the Pn, plant height, root length, and aboveground biomass could reflect the low-N tolerance of sorghum varieties more intuitively, and be used as the screening indexes for low-N tolerance in *sorghum* [23].In this study, photosynthetic index and antioxidant enzyme activities were considered critical physiological indexes for evaluating the ability of oat to tolerate low N.

Photosynthesis is the process of converting CO_2_ into glucose using light energy, and it is the initial source of material metabolism and energy conversion in crops. Photosynthesis is regulated by environmental factors, and under environmental stress, the rate of photosynthesis decreases, which is related to stomatal conductance (Gsf), photosynthetic rate (Pn), and internal enzyme activity [35,36]. In this experiment, the low-N stress treatment not only reduced the stomatal conductance of oat, but also affected the leaf intercellular CO_2_ concentration, suggesting that stomatal limitation under low-N stress may be a factor contributing to the decrease in photosynthetic rate of oat leaves. In addition, the Ci and Gs of the five oat germplasm differed in the magnitude of decrease under low-N stress, and the magnitude of decrease of the low-N intolerant germplasm “ZNY256” was larger than that of the low-N tolerant germplasm “Qingyongjiu 035”, which led to a significantly higher photosynthetic rate of the low-N tolerant germplasm “Qingyongjiu 035” than that of the low-N tolerant germplasm “Qingyongjiu 035” under low-N stress. As a result, the photosynthetic rate of the low-nitrogen-tolerant oat germplasm “Qingyongjiu 035” was significantly higher than that of the low-nitrogen-intolerant germplasm “ZNY256” under low nitrogen stress. Since maintaining a relatively high leaf photosynthetic rate is important for maintaining normal plant growth when plants are subjected to low-nitrogen stress, it can be assumed that the significantly higher leaf photosynthetic rate of the low-nitrogen-tolerant germplasm than that of the low-nitrogen-intolerant germplasm under low-nitrogen conditions is one of the reasons for the high low-nitrogen-tolerance ability. In addition, under low nitrogen stress, the transpiration rate and water use efficiency of the low-nitrogen-tolerant germplasm “qing permanent 035” changed less than those of the normal nitrogen treatment, whereas those of the low-nitrogen-intolerant germplasm“ZNY256”, which may also be a factor affecting photosynthesis, and maybe the reason for the higher photosynthetic rate. This may also be an important factor affecting photosynthesis.

Relevant studies have shown that when plants are subjected to adversity stress, they will adapt to the adversity by regulating a series of physiological and biochemical responses, among which the content of osmoregulatory substances can be used as one of the indicators for evaluating plant resistance [37]. The increase in soluble sugar (SS) content can maintain the water absorption capacity of plant leaves and delay leaf senescence [38]. In low nitrogen stress, the lack of external nitrogen leads to too few nitrogen compounds synthesized by its absorption and fewer proteins can be synthesized, and the lack of nitrogen causes the osmoregulatory substances of the plant itself to be altered [39]. The results of this study showed that the low nitrogen stress treatment increased the soluble sugar content of oat leaves, but the magnitude of the increase varied, and the soluble sugar content of the low nitrogen tolerant oat germplasm increased less than that of the low nitrogen intolerant germplasm in the leaves. This suggests that compared with the non-low-nitrogen-tolerant oat germplasm, the low-nitrogen-tolerant oat germplasm transferred more soluble sugars to the roots of the plant to satisfy the normal growth of the root system, which was favorable for it to absorb more nitrogen from the low-nitrogen environment.

When plants are in an adverse environment, the dynamic balance between the production and removal of reactive oxygen species (ROS) in plant cells will be disrupted, leading to an increase in the content of ROS [40], which mainly includes superoxide anion (O^2−^) and hydrogen peroxide (H_2_O_2_), and excessive ROS will cause damage to the membrane lipid system of the cell membrane [41]. In this study, it was found that reactive oxygen species (ROS) were accumulated in large quantities in all oat materials under low-nitrogen treatment (Fig. 2), and the accumulation varied among different varieties. The results of the significant increase in ROS content in this experiment suggest that low nitrogen stress causes damage to the membrane lipid system of oat, leading to an increase in free radicals in oats, which results in oxidative damage to oats. In addition, the oxidative response of oats under low nitrogen stress also indicated that their antioxidant capacity was weakened, which might be related to the activity of antioxidant enzymes. The ability of oat cells to cope with reactive oxygen species-induced damage to the membrane system under low-N stress is a crucial feature of its low-N tolerance physiology. In addition, antioxidant enzymes also play essential roles in response to other stresses such as drought, salinity, and high and low temperatures, suggesting similarities in the physiological mechanisms of resistance to low-N stress and other stresses. Varieties with strong resistance to various adversities in oat also showed strong low-N tolerance. In this study, we measured and analyzed the relevant physiological indexes of five oat resources under low-N conditions and clarified the physiological characteristics that are closely related to the low-N tolerance ability of oat, which provides a reference for the rapid screening and breeding of low-N-tolerant oat germplasm resources and lays a foundation for the cultivation of low-N-tolerant varieties of oat.

## 5 Conclusion

Low-N stress significantly affected the physiological and biochemical characteristics of oat seedlings. The antioxidant enzyme activities, osmoregulatory substances and MDA contents of oats under low-N stress were significantly greater than those of the control, and the trends of these varied depending on the materials used for the test. Meanwhile, net photosynthetic rate, N metabolism-related enzyme activities and SS content all decreased when subjected to low-N stress. Photosynthetic index and antioxidant enzyme activity can be used as indicators for the comprehensive evaluation of low-N tolerance in the seedling stage of oats. Among the five test materials, Qingyongjiu 035 had the strongest low-N tolerance, and ZNY256 had the weakest low-N tolerance. In this study, we investigated the physiological response mechanism of oat under low-N conditions, and found a theoretical basis for research on the mechanism of low-N tolerance in oat at a later stage, as well as a reference for the selection and breeding of excellent low-N-tolerant forage grass germplasm.

## CRediT authorship contribution statement

**Jing Pan:** Writing – review & editing, Writing – original draft, Software, Project administration, Methodology, Formal analysis, Data curation. **Zeliang Ju:** Writing – original draft. **Xiang Ma:** Software, Methodology. **Lianxue Duan:** Data curation. **Zhifeng Jia:** Writing – review & editing, Writing – original draft, Supervision.

## Data availability statement

All data used in this study are included in the article.

## Funding sources

This work was financially supported by 10.13039/501100012579Qinghai Natural Science Foundation Program-Innovation Team (2022-ZJ-902).

## Declaration of competing interest

The authors declare that they have no known competing financial interests or personal relationships that could have appeared to influence the work reported in this paper.
